# 4-Chloro-*N*′-[(*Z*)-4-nitro­benzyl­idene]benzohydrazide monohydrate

**DOI:** 10.1107/S160053680802446X

**Published:** 2008-08-06

**Authors:** Hoong-Kun Fun, P. S. Patil, Jyothi N. Rao, B. Kalluraya, Suchada Chantrapromma

**Affiliations:** a X-ray Crystallography Unit, School of Physics, Universiti Sains Malaysia, 11800 USM, Penang, Malaysia; b Department of Studies in Physics, Mangalore University, Mangalagangotri, Mangalore 574 199, India; c Department of Studies in Chemistry, Mangalore University, Mangalagangotri, Mangalore 574 199, India; d Crystal Materials Research Unit, Department of Chemistry, Faculty of Science, Prince of Songkla University, Hat-Yai, Songkhla 90112, Thailand

## Abstract

In the title compound, C_14_H_10_ClN_3_O_3_·H_2_O, the benzohydrazide group is not planar and the mol­ecule exists in a *cis* configuration with respect to the methyl­idene unit. The dihedral angle between the two substituted benzene rings is 38.7 (3)°. In the crystal structure, mol­ecules are linked by O—H⋯O, O—H⋯N and N—H⋯O hydrogen bonds into a two-dimensional network parallel to the  (100) plane. The crystal structure is further stabilized by weak C—H⋯O inter­actions.

## Related literature

For bond-length data, see: Allen *et al.* (1987 ▶). For background to the activities of hydrazones, see, for example: Bedia *et al.* (2006 ▶); Rollas & Kouçoukgouzel (2007 ▶).
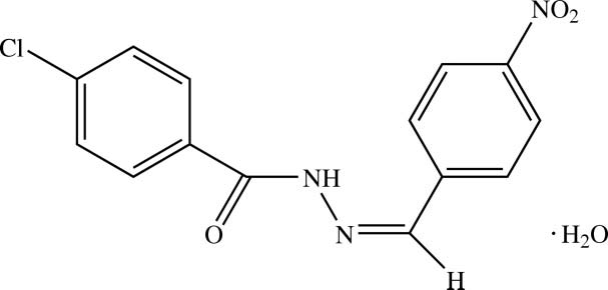


## Experimental

### 

#### Crystal data

C_14_H_10_ClN_3_O_3_·H_2_O
*M* *_r_* = 321.72
Monoclinic, 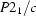

*a* = 16.3049 (8) Å
*b* = 6.8783 (4) Å
*c* = 12.7209 (7) Å
β = 104.122 (4)°
*V* = 1383.53 (13) Å^3^
*Z* = 4
Mo *K*α radiation
μ = 0.30 mm^−1^
*T* = 100.0 (1) K
0.38 × 0.21 × 0.10 mm

#### Data collection

Bruker SMART APEX2 CCD diffractometer
Absorption correction: multi-scan (*SADABS*; Bruker, 2005 ▶) *T*_min_ = 0.896, *T*_max_ = 0.971
14031 measured reflections
3172 independent reflections
2558 reflections with *I* > 2σ(*I*)
*R*_int_ = 0.044

#### Refinement

*R*[*F* ^2^ > 2σ(*F* ^2^)] = 0.086
*wR*(*F* ^2^) = 0.239
*S* = 1.13
3172 reflections
199 parameters
H-atom parameters constrained
Δρ_max_ = 1.40 e Å^−3^
Δρ_min_ = −0.46 e Å^−3^

### 

Data collection: *APEX2* (Bruker, 2005 ▶); cell refinement: *APEX2*; data reduction: *SAINT* (Bruker, 2005 ▶); program(s) used to solve structure: *SHELXTL* (Sheldrick, 2008 ▶); program(s) used to refine structure: *SHELXTL*; molecular graphics: *SHELXTL*; software used to prepare material for publication: *SHELXTL* and *PLATON* (Spek, 2003 ▶).

## Supplementary Material

Crystal structure: contains datablocks global, I. DOI: 10.1107/S160053680802446X/hb2764sup1.cif

Structure factors: contains datablocks I. DOI: 10.1107/S160053680802446X/hb2764Isup2.hkl

Additional supplementary materials:  crystallographic information; 3D view; checkCIF report

## Figures and Tables

**Table 1:** Hydrogen-bond geometry (Å, °)

*D*—H⋯*A*	*D*—H	H⋯*A*	*D*⋯*A*	*D*—H⋯*A*
O1*W*—H1*W*⋯O3^i^	0.88	1.93	2.794 (5)	168
O1*W*—H2*W*⋯O3^ii^	0.89	2.29	2.898 (5)	126
O1*W*—H2*W*⋯N2^ii^	0.89	2.32	3.185 (5)	163
N1—H1*N*1⋯O1*W*	0.85	2.04	2.818 (5)	151
C2—H2*A*⋯O1^iii^	0.93	2.40	3.329 (6)	176
C14—H14*A*⋯O1*W*^iv^	0.93	2.51	3.322 (6)	146

Symmetry codes: (i) 
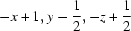
; (ii) 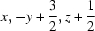; (iii) 
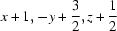
; (iv) 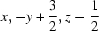.

**Fig. 1.**
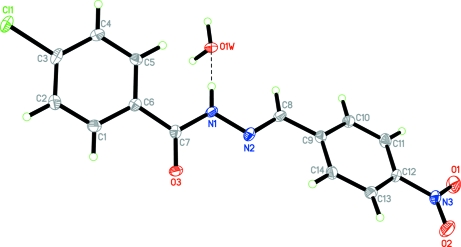
The molecular structure of (I), showing 50% probability displacement ellipsoids for the non-hydrogen atoms. The N—H···O hydrogen bond is shown as a dashed line.

**Fig. 2.**
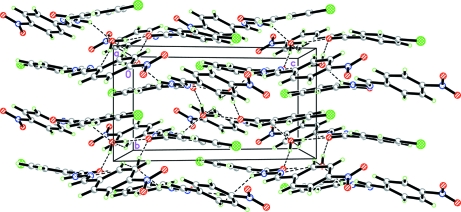
The packing diagram of (I), viewed along the a axis. Hydrogen bonds are shown as dashed lines.

## supplementary crystallographic information

### Comment

Hydrazones have been demonstrated to possess antimicrobial, anticonvulsant, analgesic, antiinflammatory, antiplatelet, antitubercular, anticancer and antitumor activities (e.g. Bedia *et al.*, 2006). Hydrazones possessing an azometine –NHN=CH– proton constitute an important class of compounds for new drug development. Many researchers have therefore synthesized these compounds as target structures and evaluated their biological activities. These observations have served as guides for the development of new hydrazones that possess varied biological activities.  These compounds are synthesized by heating the appropriate substituted hydrazines/hydrazides with aldehydes and ketones in solvents like ethanol, methanol, tetrahydrofuran, butanol, glacial acetic acid, ethanol-glacial and acetic acid. Another synthetic route for the synthesis of hydrazones is the coupling of aryldiazonium salts with active hydrogen compounds (Rollas & Kοuçοukgοuzel, 2007).

In the structure of the title compound (I) (Fig. 1), the molecule exist in a *cis*-configuration with respect to the methylidene unit (C8=N2). The dihedral angle between the two substituted benzene rings is 38.7 (3)°. In the 4-nitrophenyl unit, the nitro group is slightly twisted from the mean plane of the C9–C14 ring with the torsion angles O1–N3–C12–C13 = 174.9 (5)° and O2–N3–C12–C13 = -4.4 (8)°. The benzohydrazide moiety (N1/N2/O3/C1–C7) is not planar as indicated by the interplanar angle between the N1/N2/O3/C7 plane and C1–C6 pheny ring of 17.2 (3)°. The mean plane through N1/N2/C8/C9 plane makes the dihedral angle of 9.1 (5)° with the N1/N2/O3/C7 plane. The orientation of the benzohydrazide with respect to methylidine unit can be indicated by the torsion C7–N1–N2–C8 of 174.0 (5)°. The bond distances and angles are in normal ranges (Allen *et al.*, 1987).

The water molecule is involved in O—H···O, O—H···N and N—H···O hydrogen bonds (Table 1). These hydrogen bonds linked the molecules into two dimensional networks parallel to the (100) plane as shown in Fig. 2. The crystal is further stabilized by weak C—H···O interactions (Table 1).

### Experimental

The title compound was prepared by refluxing 4-chlorophenyl hydrazide (0.01 mol), 4-nitro benzaldehyde (0.01 mol) in ethanol (30 ml) and 3 drops of concentrated sulfuric acid for 3 hrs. Excess ethanol was removed from the reaction mixture under reduced pressure. The solid product obtained was filtered, washed with water and dried. Colorless needles of (I) were obtained from an ethanol solution by slow evaporation (Yield 53%), *M*.p. 488 K.

### Refinement

All the H atoms were placed in calculated positions (N—H = 0.85Å, O—H = 0.88-0.89Å, C—H = 0.93Å) and refined as riding with U_iso_(H) = 1.2U_eq_(carrier). The highest residual electron density peak is 1.88 Å from H13A and the deepest hole is 0.87 Å from C7.

### Crystal data

**Table:**

C_14_H_10_ClN_3_O_3_·H_2_O	*F*_000_ = 664
*M**_r_* = 321.72	*D*_x_ = 1.545 Mg m^−^^3^
Monoclinic, *P*2_1_/*c*	Melting point: 488 K
Hall symbol: -P 2ybc	Mo *K*α radiation λ = 0.71073 Å
*a* = 16.3049 (8) Å	Cell parameters from 3172 reflections
*b* = 6.8783 (4) Å	θ = 1.3–27.5º
*c* = 12.7209 (7) Å	µ = 0.30 mm^−^^1^
β = 104.122 (4)º	*T* = 100.0 (1) K
*V* = 1383.53 (13) Å^3^	Needle, colorless
*Z* = 4	0.38 × 0.21 × 0.10 mm

### Data collection

**Table:**

Bruker SMART APEX2 CCD diffractometer	3172 independent reflections
Radiation source: fine-focus sealed tube	2558 reflections with *I* > 2σ(*I*)
Monochromator: graphite	*R*_int_ = 0.044
Detector resolution: 8.33 pixels mm^-1^	θ_max_ = 27.5º
*T* = 100.0(1) K	θ_min_ = 1.3º
ω scans	*h* = −21→21
Absorption correction: Multi-scan(SADABS; Bruker, 2005)	*k* = −7→8
*T*_min_ = 0.896, *T*_max_ = 0.971	*l* = −16→16
14031 measured reflections	

### Refinement

**Table:**

Refinement on *F*^2^	Secondary atom site location: difference Fourier map
Least-squares matrix: full	Hydrogen site location: inferred from neighbouring sites
*R*[*F*^2^ > 2σ(*F*^2^)] = 0.086	H-atom parameters constrained
*wR*(*F*^2^) = 0.239	*w* = 1/[σ^2^(*F*_o_^2^) + (0.0417*P*)^2^ + 15.1986*P*] where *P* = (*F*_o_^2^ + 2*F*_c_^2^)/3
*S* = 1.13	(Δ/σ)_max_ < 0.001
3172 reflections	Δρ_max_ = 1.40 e Å^−^^3^
199 parameters	Δρ_min_ = −0.46 e Å^−^^3^
Primary atom site location: structure-invariant direct methods	Extinction correction: none

### Special details

**Table:**

Experimental. The low-temperature data was collected with the Oxford Cyrosystem Cobra low-temperature attachment.
Geometry. All e.s.d.'s (except the e.s.d. in the dihedral angle between two l.s. planes) are estimated using the full covariance matrix. The cell e.s.d.'s are taken into account individually in the estimation of e.s.d.'s in distances, angles and torsion angles; correlations between e.s.d.'s in cell parameters are only used when they are defined by crystal symmetry. An approximate (isotropic) treatment of cell e.s.d.'s is used for estimating e.s.d.'s involving l.s. planes.
Refinement. Refinement of *F*^2^ against ALL reflections. The weighted *R*-factor *wR* and goodness of fit *S* are based on *F*^2^, conventional *R*-factors *R* are based on *F*, with *F* set to zero for negative *F*^2^. The threshold expression of *F*^2^ > σ(*F*^2^) is used only for calculating *R*-factors(gt) *etc*. and is not relevant to the choice of reflections for refinement. *R*-factors based on *F*^2^ are statistically about twice as large as those based on *F*, and *R*- factors based on ALL data will be even larger.

### Fractional atomic coordinates and isotropic or equivalent isotropic displacement parameters (Å^2^)

**Table:**

	*x*	*y*	*z*	*U*_iso_*/*U*_eq_	
Cl1	0.79974 (8)	0.8946 (2)	0.59885 (10)	0.0211 (3)	
O1	−0.0852 (2)	0.7494 (7)	−0.1492 (3)	0.0312 (10)	
O2	−0.0194 (2)	0.9231 (7)	−0.2447 (3)	0.0306 (10)	
O3	0.5057 (2)	0.8432 (6)	0.1303 (3)	0.0167 (8)	
N1	0.4189 (2)	0.8116 (6)	0.2433 (3)	0.0146 (8)	
H1N1	0.4111	0.7946	0.3065	0.017*	
N2	0.3494 (3)	0.8265 (6)	0.1565 (3)	0.0154 (9)	
N3	−0.0222 (3)	0.8347 (7)	−0.1617 (3)	0.0196 (9)	
C1	0.6514 (3)	0.8141 (8)	0.3030 (4)	0.0172 (10)	
H1A	0.6578	0.7885	0.2336	0.021*	
C2	0.7224 (3)	0.8303 (8)	0.3878 (4)	0.0184 (10)	
H2A	0.7761	0.8149	0.3762	0.022*	
C3	0.7118 (3)	0.8698 (8)	0.4901 (4)	0.0173 (10)	
C4	0.6319 (3)	0.8910 (8)	0.5090 (4)	0.0173 (10)	
H4A	0.6259	0.9173	0.5785	0.021*	
C5	0.5614 (3)	0.8724 (8)	0.4236 (4)	0.0153 (10)	
H5A	0.5077	0.8847	0.4359	0.018*	
C6	0.5702 (3)	0.8354 (7)	0.3196 (4)	0.0143 (10)	
C7	0.4969 (3)	0.8287 (7)	0.2234 (4)	0.0141 (9)	
C8	0.2778 (3)	0.7923 (8)	0.1770 (4)	0.0166 (10)	
H8A	0.2749	0.7564	0.2465	0.020*	
C9	0.2003 (3)	0.8100 (8)	0.0902 (4)	0.0152 (10)	
C10	0.1241 (3)	0.7367 (8)	0.1067 (4)	0.0181 (11)	
H10A	0.1230	0.6841	0.1737	0.022*	
C11	0.0507 (3)	0.7417 (8)	0.0248 (4)	0.0193 (11)	
H11A	0.0005	0.6890	0.0346	0.023*	
C12	0.0543 (3)	0.8281 (8)	−0.0724 (4)	0.0171 (10)	
C13	0.1275 (3)	0.9111 (8)	−0.0900 (4)	0.0165 (10)	
H13A	0.1272	0.9735	−0.1549	0.020*	
C14	0.2009 (3)	0.8985 (8)	−0.0080 (4)	0.0168 (10)	
H14A	0.2510	0.9496	−0.0187	0.020*	
O1W	0.3845 (2)	0.6173 (6)	0.4228 (3)	0.0196 (8)	
H1W	0.4181	0.5223	0.4141	0.029*	
H2W	0.3862	0.6256	0.4931	0.029*	

### Atomic displacement parameters (Å^2^)

**Table:**

	*U*^11^	*U*^22^	*U*^33^	*U*^12^	*U*^13^	*U*^23^
Cl1	0.0164 (6)	0.0226 (7)	0.0194 (6)	−0.0002 (5)	−0.0049 (4)	−0.0009 (5)
O1	0.0116 (17)	0.049 (3)	0.032 (2)	−0.0057 (18)	0.0034 (15)	0.003 (2)
O2	0.022 (2)	0.046 (3)	0.0199 (19)	−0.0012 (19)	−0.0013 (15)	0.0072 (19)
O3	0.0168 (16)	0.023 (2)	0.0106 (15)	−0.0028 (15)	0.0035 (12)	−0.0020 (14)
N1	0.0144 (19)	0.019 (2)	0.0097 (17)	−0.0017 (17)	0.0010 (14)	−0.0001 (17)
N2	0.0141 (19)	0.015 (2)	0.0153 (19)	−0.0017 (16)	−0.0010 (15)	−0.0012 (17)
N3	0.0113 (19)	0.027 (3)	0.019 (2)	0.0000 (18)	0.0008 (16)	0.0010 (19)
C1	0.020 (2)	0.018 (3)	0.015 (2)	0.001 (2)	0.0050 (18)	0.000 (2)
C2	0.014 (2)	0.019 (3)	0.022 (2)	0.001 (2)	0.0038 (19)	0.000 (2)
C3	0.016 (2)	0.015 (3)	0.017 (2)	−0.0024 (19)	−0.0034 (18)	0.002 (2)
C4	0.020 (2)	0.018 (3)	0.013 (2)	−0.001 (2)	0.0028 (18)	−0.001 (2)
C5	0.016 (2)	0.016 (2)	0.015 (2)	0.0005 (19)	0.0049 (17)	−0.0023 (19)
C6	0.015 (2)	0.011 (2)	0.016 (2)	−0.0011 (18)	0.0024 (17)	−0.0006 (19)
C7	0.013 (2)	0.011 (2)	0.018 (2)	0.0008 (18)	0.0025 (18)	−0.0003 (19)
C8	0.018 (2)	0.019 (3)	0.012 (2)	0.000 (2)	0.0015 (18)	0.000 (2)
C9	0.013 (2)	0.016 (2)	0.015 (2)	−0.0001 (19)	0.0026 (17)	−0.001 (2)
C10	0.017 (2)	0.023 (3)	0.015 (2)	0.000 (2)	0.0055 (18)	0.005 (2)
C11	0.014 (2)	0.022 (3)	0.022 (2)	−0.001 (2)	0.0044 (19)	0.003 (2)
C12	0.013 (2)	0.022 (3)	0.015 (2)	0.003 (2)	0.0000 (17)	0.001 (2)
C13	0.015 (2)	0.019 (3)	0.014 (2)	−0.001 (2)	0.0030 (18)	0.000 (2)
C14	0.013 (2)	0.019 (3)	0.018 (2)	0.0011 (19)	0.0031 (18)	−0.001 (2)
O1W	0.0201 (17)	0.028 (2)	0.0111 (15)	0.0008 (16)	0.0047 (13)	−0.0007 (15)

### Geometric parameters (Å, °)

**Table:**

Cl1—C3	1.741 (5)	C5—C6	1.389 (7)
O1—N3	1.226 (6)	C5—H5A	0.9300
O2—N3	1.228 (6)	C6—C7	1.488 (6)
O3—C7	1.232 (6)	C8—C9	1.466 (6)
N1—C7	1.361 (6)	C8—H8A	0.9300
N1—N2	1.379 (5)	C9—C14	1.391 (7)
N1—H1N1	0.8525	C9—C10	1.404 (7)
N2—C8	1.279 (6)	C10—C11	1.383 (7)
N3—C12	1.469 (6)	C10—H10A	0.9300
C1—C2	1.380 (7)	C11—C12	1.387 (7)
C1—C6	1.399 (7)	C11—H11A	0.9300
C1—H1A	0.9300	C12—C13	1.390 (7)
C2—C3	1.382 (7)	C13—C14	1.384 (7)
C2—H2A	0.9300	C13—H13A	0.9300
C3—C4	1.388 (7)	C14—H14A	0.9300
C4—C5	1.381 (6)	O1W—H1W	0.8771
C4—H4A	0.9300	O1W—H2W	0.8898
			
C7—N1—N2	117.9 (4)	O3—C7—N1	121.2 (4)
C7—N1—H1N1	123.2	O3—C7—C6	122.0 (4)
N2—N1—H1N1	118.9	N1—C7—C6	116.8 (4)
C8—N2—N1	115.9 (4)	N2—C8—C9	119.6 (4)
O1—N3—O2	123.8 (4)	N2—C8—H8A	120.2
O1—N3—C12	117.7 (4)	C9—C8—H8A	120.2
O2—N3—C12	118.5 (4)	C14—C9—C10	119.4 (4)
C2—C1—C6	121.2 (5)	C14—C9—C8	121.3 (4)
C2—C1—H1A	119.4	C10—C9—C8	119.3 (4)
C6—C1—H1A	119.4	C11—C10—C9	120.9 (5)
C1—C2—C3	118.6 (5)	C11—C10—H10A	119.6
C1—C2—H2A	120.7	C9—C10—H10A	119.6
C3—C2—H2A	120.7	C10—C11—C12	117.7 (5)
C2—C3—C4	121.4 (4)	C10—C11—H11A	121.1
C2—C3—Cl1	120.0 (4)	C12—C11—H11A	121.1
C4—C3—Cl1	118.6 (4)	C11—C12—C13	123.0 (4)
C5—C4—C3	119.3 (5)	C11—C12—N3	119.3 (4)
C5—C4—H4A	120.3	C13—C12—N3	117.7 (4)
C3—C4—H4A	120.3	C14—C13—C12	118.1 (5)
C4—C5—C6	120.4 (5)	C14—C13—H13A	120.9
C4—C5—H5A	119.8	C12—C13—H13A	120.9
C6—C5—H5A	119.8	C13—C14—C9	120.6 (5)
C5—C6—C1	119.0 (4)	C13—C14—H14A	119.7
C5—C6—C7	122.7 (4)	C9—C14—H14A	119.7
C1—C6—C7	118.2 (4)	H1W—O1W—H2W	107.9
			
C7—N1—N2—C8	174.0 (5)	N1—N2—C8—C9	178.3 (4)
C6—C1—C2—C3	−0.5 (8)	N2—C8—C9—C14	−12.5 (8)
C1—C2—C3—C4	0.8 (8)	N2—C8—C9—C10	167.7 (5)
C1—C2—C3—Cl1	−179.2 (4)	C14—C9—C10—C11	3.6 (8)
C2—C3—C4—C5	−0.1 (8)	C8—C9—C10—C11	−176.7 (5)
Cl1—C3—C4—C5	179.8 (4)	C9—C10—C11—C12	−2.4 (8)
C3—C4—C5—C6	−0.8 (8)	C10—C11—C12—C13	−1.0 (8)
C4—C5—C6—C1	1.1 (8)	C10—C11—C12—N3	179.5 (5)
C4—C5—C6—C7	−175.3 (5)	O1—N3—C12—C11	−5.5 (8)
C2—C1—C6—C5	−0.4 (8)	O2—N3—C12—C11	175.2 (5)
C2—C1—C6—C7	176.2 (5)	O1—N3—C12—C13	174.9 (5)
N2—N1—C7—O3	−5.1 (7)	O2—N3—C12—C13	−4.4 (8)
N2—N1—C7—C6	173.0 (4)	C11—C12—C13—C14	3.1 (8)
C5—C6—C7—O3	162.0 (5)	N3—C12—C13—C14	−177.3 (5)
C1—C6—C7—O3	−14.4 (8)	C12—C13—C14—C9	−1.9 (8)
C5—C6—C7—N1	−16.1 (7)	C10—C9—C14—C13	−1.3 (8)
C1—C6—C7—N1	167.5 (5)	C8—C9—C14—C13	178.9 (5)

### Hydrogen-bond geometry (Å, °)

**Table:**

*D*—H···*A*	*D*—H	H···*A*	*D*···*A*	*D*—H···*A*
O1W—H1W···O3^i^	0.88	1.93	2.794 (5)	168
O1W—H2W···O3^ii^	0.89	2.29	2.898 (5)	126
O1W—H2W···N2^ii^	0.89	2.32	3.185 (5)	163
N1—H1N1···O1W	0.85	2.04	2.818 (5)	151
C2—H2A···O1^iii^	0.93	2.40	3.329 (6)	176
C14—H14A···O1W^iv^	0.93	2.51	3.322 (6)	146

Symmetry codes: (i) −*x*+1, *y*−1/2, −*z*+1/2; (ii) *x*, −*y*+3/2, *z*+1/2;  (iii) *x*+1, −*y*+3/2, *z*+1/2; (iv) *x*, −*y*+3/2, *z*−1/2.

## References

[bb1] Allen, F. H., Kennard, O., Watson, D. G., Brammer, L., Orpen, A. G. & Taylor, R. (1987). *J. Chem. Soc. Perkin Trans. 2*, pp. S1–S19.

[bb2] Bedia, K.-K., Elçin, O., Seda, U., Fatma, K., Nathaly, S., Sevim, R. & Dimoglo, A. (2006). *Eur. J. Med. Chem.* **41**, 1253–1261. 10.1016/j.ejmech.2006.06.009 16919372

[bb3] Bruker (2005). *APEX2*, *SAINT* and *SADABS* Bruker AXS Inc., Madison, Wisconsin, USA.

[bb4] Rollas, S. & Kouçoukguzel, Ş. G. (2007). *Molecules*, **12**, 1910–1939.

[bb5] Sheldrick, G. M. (2008). *Acta Cryst.* A**64**, 112–122. 10.1107/S0108767307043930 18156677

[bb6] Spek, A. L. (2003). *J. Appl. Cryst.* **36**, 7–13.

